# Occupational and environmental risk factors of idiopathic pulmonary fibrosis: a systematic review and meta-analyses

**DOI:** 10.1038/s41598-021-81591-z

**Published:** 2021-03-02

**Authors:** Yeonkyung Park, Chiwon Ahn, Tae-Hyung Kim

**Affiliations:** 1 Division of Pulmonary and Critical Care Medicine, Department of Internal Medicine, Veterans Health Service Medical Center, Seoul, South Korea; 2 Department of Emergency Medicine, College of Medicine, Chung-Ang University, Seoul, South Korea; 3 Division of Pulmonary and Critical Care Medicine, Department of Internal Medicine, College of Medicine, Hanyang University Guri Hospital, 153, Gyeongchun-ro, Guri-si, Gyeonggi-do 11923 South Korea

**Keywords:** Environmental sciences, Environmental social sciences, Health occupations

## Abstract

Idiopathic pulmonary fibrosis (IPF) is a chronic, progressive, fibrosing interstitial lung disease of unknown cause. It has a high risk of rapid progression and mortality. We conducted a systematic review and meta-analysis to evaluate the risk factor of IPF. We searched Medline, Embase, and the Cochrane library from the earliest record to March, 2020. Case–control studies on occupational and environmental risk factors or on jobs with a risk of IPF were searched for. From 2490 relevant records, 12 studies were included. Any occupational or environmental exposure to metal dust (OR 1.83, 95% CI 1.15–2.91, I^2^ = 54%), wood dust (OR 1.62 5% CI 1.04–2.53, I^2^ = 5%) and pesticide (OR 2.07, 95% CI 1.24–3.45, I^2^ = 0%) were associated with an increased risk of IPF. Farming or agricultural work (OR 1.88, 95% CI 1.17–3.04, I^2^ = 67%) was also associated with an increased risk of IPF. Moreover, smoking increased IPF risk with an odds ratio of 1.39 (95% CI 1.01–1.91, I^2^ = 29%). In conclusion, metal dust, wood dust, pesticide, occupational history of farming or agriculture and ever smoking increased the risk of IPF.

## Introduction

Interstitial lung disease (ILD) causes abnormal collagen deposition by proliferation of interstitial compartments and infiltration of various inflammatory cells, and fibrotic changes. Idiopathic pulmonary fibrosis (IPF) is a special form of chronic ILD of unknown cause that occurs mainly in the lungs with increasing age and is associated with histopathological or radiological form of usual interstitial pneumonia (UIP). To diagnose IPF, other types of ILD should be ruled out, including drug-induced ILD, ILD through environmental exposure, or systemic disease-related ILD^1^. It has been reported that increasing age, genetic predisposing factors, smoking, or continuous exposure to various environmental and occupational factors can cause physical irritation and damage to the lungs^2^. IPF prevalence is higher in men and increases with age. According to a national survey in Korea, 72.4% of IPF patients are men and the average age at diagnosis is 69 years^3^.

Several studies conducted in various countries have investigated the association between occupational and environmental exposure factors, and IPF over the past decades. According to the 2015 Korean National Health and Nutrition Survey, those exposed to occupational and environmental dust were diagnosed with IPF at a younger age and had a longer period of symptomatic symptoms at the time of diagnosis. Moreover, it has been reported to be related to increase the mortality rate of IPF patients in exposed group^4^.

Case–control studies have investigated the association of IPF incidence with each of the exposure factors that can cause IPF in various countries like the UK^5–8^, the USA^9,10^, Sweden^11^, Mexico^12^, Egypt^13^, South Korea^14,15^, and Southern Europe^16^. Exposure to metal dust^5–7,9,15–17^, wood dust^6,7,11,13^, stone or sand dust^9,14^, and raising of birds or livestock and working in agriculture^6,9,13,16^ are associated with IPF incidence. Although smoking has not been established as a causative agent, it has been shown to increase the risk of IPF^18^. In 2006, Taskar and Coultas et al. reported a significant increase in risk of IPF on stone/sand/soil exposure in a meta-analysis of four papers^18^. Additionally, a survey of occupational burdens in benign respiratory diseases, jointly conducted by the American Respiratory Society and the European Respiratory Society in 2019, revealed that exposure to silica, wood dust, metal dust, agricultural dust, and vapors, gas, dust, or fumes increased the risk of IPF^19^.

Workers in agriculture^9,13,16^, livestock industry^9^, chemical, petrochemical industry^13^, woodworking industry^13^, and steel industry^16^ had higher risk of IPF. On the other hand, a study on the occupational burden in benign respiratory diseases conducted by the American Respiratory Society and the European Respiratory Society showed no significant association between IPF incidence with these specific occupational groups^19^.

IPF is ILD due to unknown causes. Case–control studies have been conducted on the relationship between various occupational and environmental risk factors and IPF. However, the results of such studies are inconsistent. Therefore, in this study, through systematic literature review, the effects of occupational and environmental exposure factors on IPF incidence and the influence of the individual’s past or present occupation and IPF incidence were investigated. The relationship between smoking history and the incidence of IPF was also investigated.

## Results

### Search result

**Figure 1:**
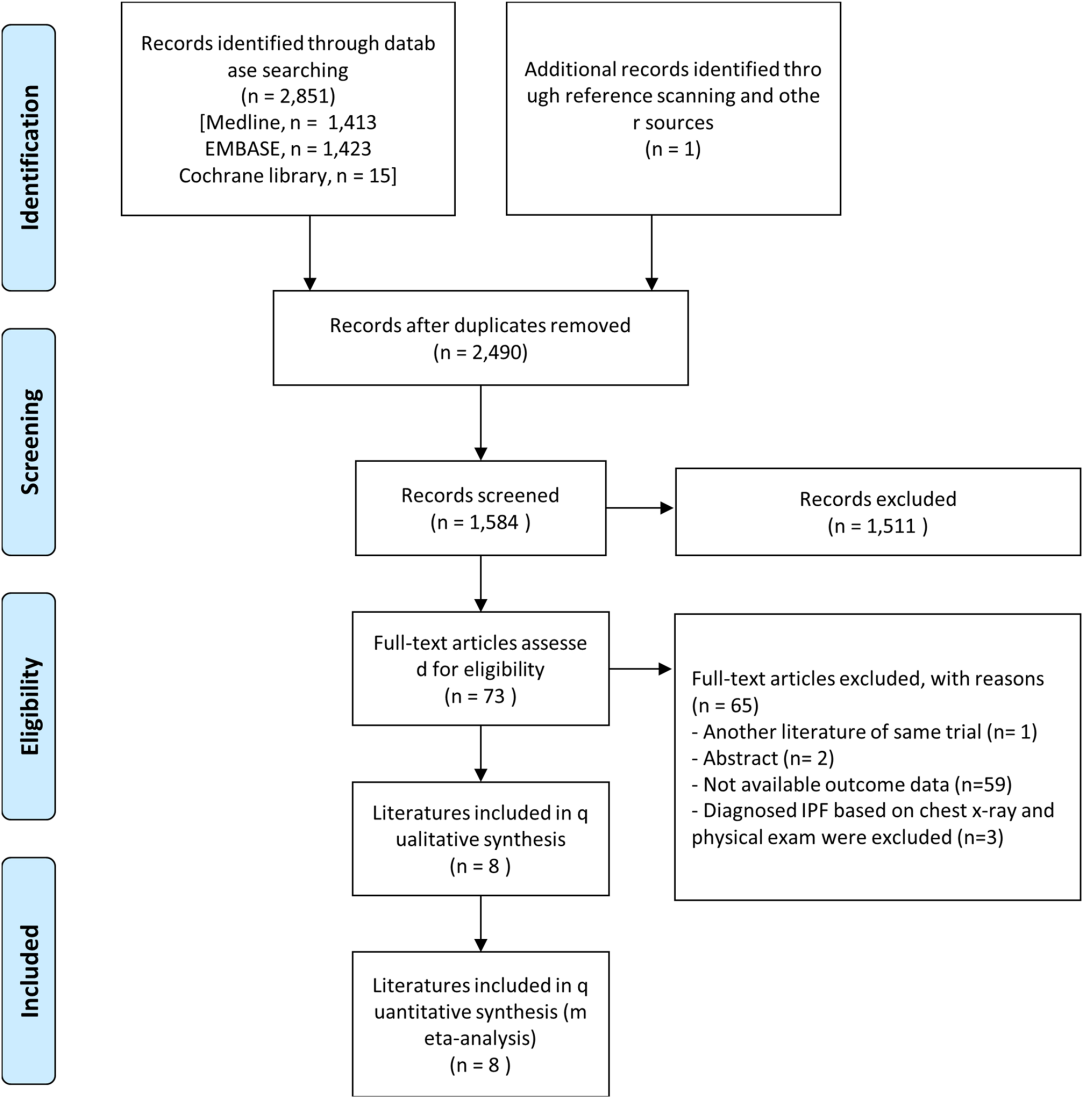
PRISMA (preferred reporting items for systematic reviews and meta-analyses) flow diagram.

In total, 2852 studies were included: Medline (n = 1413); Embase (n = 1423); the Cochrane Library (n = 15); additionally identified in the literature review process (n = 1). By reviewing the title and the abstract, a total of 73 papers were analyzed, excluding documents that did not meet the purpose of this study. In total, 8 case–control studies were included. Fifty-nine articles with unclear data or not with a case–control study design were excluded. Two abstracts, one of which was later published as an article, were excluded; another abstract sharing the same case–control cohort was excluded. Three case–control studies diagnosed IPF based on chest X-ray and physical exam were excluded. In total, 8 case–control studies were included (Fig. 1).

### Characteristics of studies and participants included

Table 1 summarizes the characteristics of the eight studies and participants included. The studies were conducted in various countries, such as the United States, Japan, Sweden, Southern Europe, Mexico, Egypt, and South Korea. In four of the eight studies, the non-IPF control group included healthy adults from the community or hospital patients without lung disease including IPF. However, in their non-IPF control group Miyake et al. included patients who visited the hospital with acute bacterial pneumonia or cold; Garcia-Sancho et al., with asthma, chronic obstructive pulmonary disease (COPD), lung cancer, and otorhinolaryngology problems; Awadalla et al., with chest infection, bronchial asthma, COPD, bronchiectasis, pulmonary embolism, and bronchogenic carcinoma; Koo et al., with pulmonary tuberculosis and community acquired pneumonia. In two studies, the survey was conducted using organized questions through a self-reporting questionnaire or a phone call or mail. All studies analyzed occupational and environmental exposure risk factors and five studies analyzed occupation types. In the included studies, the mean age of the subjects ranged between 50 and 75 years, and in four of the eight studies, the age-sex distribution between the IPF patient group and the non-IPF control group was matched without statistically significant differences. Four studies did not provide data or did not match the age and sex proportions between the two groups. High rates of smoking were observed in the IPF patient group, except for the study by Awadalla et al. which matched smoking history in advance.

**Table 1:** Characteristics of Included studies.

Study	Country	Settings of case–control study	Sources of non-IPF subjects	Methods	Subjects numbers		Age		Males (%)		Ever smoker (%)		Diagnosis of IPF
					IPF	Non-IPF	IPF	Non-IPF	IPF	Non-IPF	IPF	Non-IPF	
Baumgartner 2000^9^	USA	Multicenter age, sex, residence matched	Community	Telephone interview	248	491	61 ± 10.4	59 ± 10.5	60	60	N/A	N/A	Clinical history, Pathologically or radiologically diagnosis
Miyake 2005^17^	Japan	Multicenter	Hospital based	Self-reporting postal questionnaire, telephone interview	102	59	< 50 (2.9%)	< 50 (3.4%)	90.2	91.5	76.3	N/A	Based on 2002 ATS/ERS diagnostic criteria
							50–59 (14.7%)	50–59 (32.3%)					
							60–69 (54.9%)	60–69 (40.7%)					
							> 70 (27.5%)	> 70 (23.7%)					
Gustafon 2007^11^	Sweden	Swedish long term oxygen registry	Community	Self-reporting postal questionnaire	140	757	N/A	N/A	N/A	46.1	N/A	N/A	Biopsy confirmed after lung transplantation
Garcia-Sancho 2010^12^	Mexico	Multicenter	Other respiratory disorders	Interview by Social Work Department	97	560	62.6 ± 11.0	62.3 ± 12.2	73.2	62.0	45.4	42.9	Based on 2000 ATS/ERS diagnostic criteria
Awadalla 2012^13^	Egypt	Multicenter age, sex, residence, smoking habits matched	Hospital based	Face to face interview	201	205	51.0 ± 10.5	50.3 ± 10.4	47.3	55.6	29.9	32.7	Based on 2002 ATS/ERS diagnostic criteria
Kim 2017^14^	South Korea	SINGLE center, age, sex matched retrospective	Community	Telephone interview with trained specialists	70	70	50–59 (17.1%)	50–59 (18.6%)	75.7	75.7	75.7	54.3	Based on 2002 ATS/ERS diagnostic criteria
							60–69 (25.7%)	60–69 (24.3%)					
							70–79 (44.3%)	70–79 (44.3%)					
							80–89 (12.9%)	80–89 (12.9%)					
Koo 2017^15^	South Korea	Multicenter age group, sex, residence matched	Other respiratory disorders	Interview with occupational physicians	78	78	69.6 ± 8.8	70.6 ± 9.5	70.5	70.5	66.7	53.8	Based on ATS/ERS/JRS/LARA 2011 diagnostic criteria
Paolocci 2018^16^	Southern Europe	Multicenter	Community	Telephone interview	69	277	75 ± 14	71 ± 13	72.5	54.2	60.9	60.3	Based on UIP pattern on CT

Ages were shown in mean ± SD, if mean age were not mentioned, the proportion of age groups were available on data.

*USA* United States of America, *N/A* not available.

### Quality assessment of studies

Among the eight studies included, five studies score less than 1 with high quality, one study in moderate quality, and two studies in low quality. In the measurement of intervention category, two studies were evaluated as “high” because the questionnaire was self-reported by postal questionnaire or telephone interview. In the confounding variables category, two studies showing differences in age or sex composition between the IPF and non-IPF groups were evaluated as “high”. In the selective outcome-reporting category, one study was evaluated as “high” because only statistically significant exposure risk factor results were mentioned (Supplementary Fig. S1).

### Occupational and environmental exposure factors and risk of IPF

**Figure 2:**
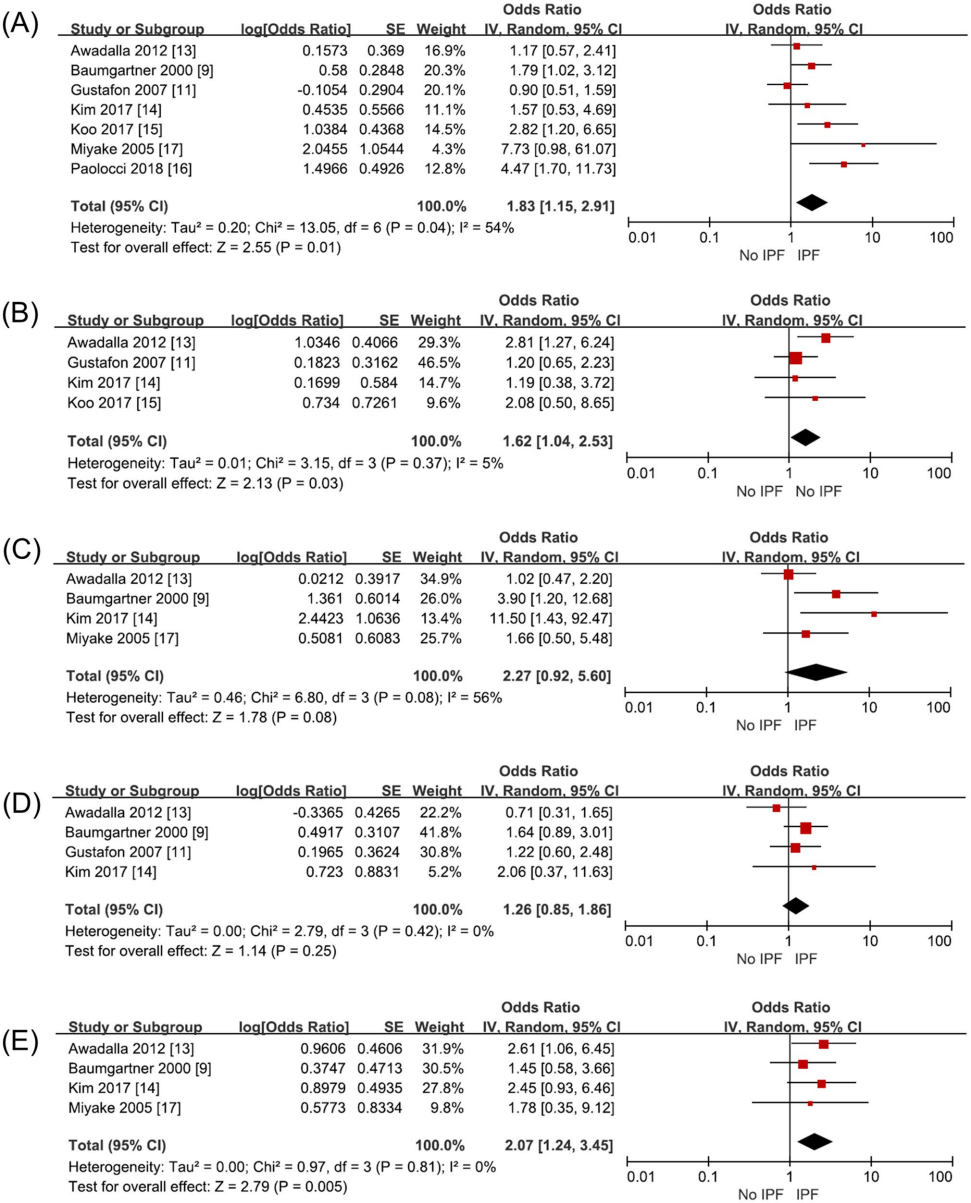
Risk of IPF in exposure to occupational and environmental risk factors compared with non-IPF subjects. (**A**) metal dust, (**B**) wood dust, (**C**) stone and sand dust, (**D**) textile dust, and (**E**) pesticide.

Seven studies (2845 subjects) investigated metal dust exposure. Three papers^9,15,16^ had increased the risk of IPF and four studies had no relationship^11,13,14,17^. Awadalla et al., Gustafon et al., Kim et al. and Paolocci et al. investigated the metal dust and metal fumes as one category. Baumgartner et al. with metal dust excluding aluminum, beryllium, and cobalt and Koo et al. investigated metal dust and fumes separately. With seven studies on analysis, metal dust increased the risk of IPF with an odds ratio of 1.83 (95% CI 1.15–2.91, p = 0.01, I^2^ = 54%) (Fig. 2A, Supplementary Table S2).

Four studies (1599 subjects) investigated wood dust exposure. Among them, Awadalla et al. investigated exposure to wood dust and to wood preservatives as one exposure factor and Gustafon et al. investigated exposure to wood dust, hardwood dust and birch into different risk factor. Exposure to wood dust statistically significantly increased the risk of IPF with an odds ratio of 1.62 (95% CI 1.04–2.53, p = 0.03, I^2^ = 5%) (Fig. 2B, Supplementary Table S2).

Four studies (1446 subjects) investigated stone/sand dust exposure. Miyake et al. and Baumgather et al. investigated stone and sand dust exposure; Awadalla et al., stone, glass, and concrete dust exposure. Kim et al. surveyed exposure to stone and sand dust containing silica. On combining all their results, the risk of IPF was not increased with an odds ratio of 2.27 (95% CI 0.92–5.60, p = 0.06, I^2^ = 56%) when exposed to stone/sand dust (Fig. 2C, Supplementary Table S2).

Four studies (2182 subjects) investigated textile dust exposure. The risk of IPF did not increase on exposure to textile dust with an odds ratio of 1.26 (95% CI 0.85–1.86, p = 0.25, I^2^ = 0%) (Fig. 2D, Supplementary Table S2).

Four studies (1446 subjects) investigated pesticide exposure, which on meta-analysis was found to increase IPF risk with an odds ratio of 2.07 (95% CI 1.24–3.45, p = 0.005, I^2^ = 0%) (Fig. 2E, Supplementary Table S2).

### Job and risk of IPF

**Figure 3:**
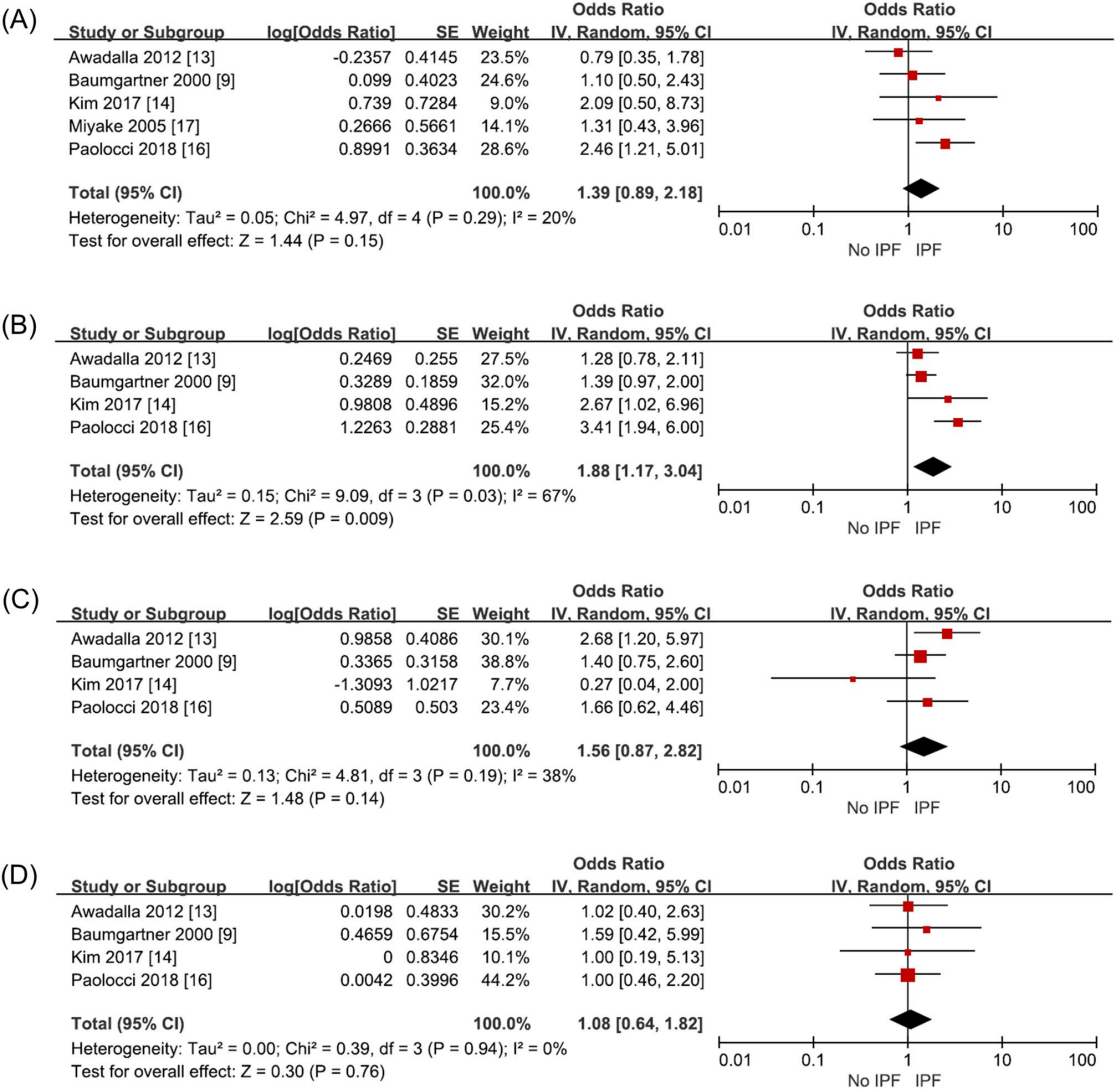
Risk of IPF in occupation compared to non-IPF controls. (**A**) building construction and demolition workers, (**B**) farming or agriculture workers, (**C**) carpentry and wood workers, and (**D**) textile making workers.

Five studies (1792 subjects) investigated exposure through working in the construction industry, including working at building construction and sites. IPF risk on such exposure increased with an odds ratio of 1.39 (95% CI 0.89–2.18, p = 0.15, I^2^ = 20%), but it was not statistically significant (Fig. 3A, Supplementary Table S3).

Four studies (1631 subjects) investigated exposure through working in the agriculture sector. Paolocci et al. classified agriculture, veterinarians, and gardeners into one occupation group. Miyake et al. unified agriculture and fisheries into one occupational category. While, Awadalla et al., separated agriculture and fisheries into different occupational categories. Therefore, the study by Miyake et al. was excluded from the analysis. On meta-analysis, exposure as agricultural workers increased IPF risk statistically significantly (OR 1.88, 95% CI 1.17–3.04, p = 0.009, I^2^ = 67%) (Fig. 3B, Supplementary Table S3). Heterogeneity was high in this analysis. When the sensitivity analysis was performed excluding Paolocci et al., the heterogeneity decreased to I^2^ = 0 (OR 1.43, 95% CI 1.08–1.90, p = 0.01) and it was as statistically significant as the previous results. This was confirmed because Paolocci et al. included veterinarians and gardeners into the same occupation group as agricultural workers, unlike the other 3 studies.

Four studies (1631 subjects) analyzed exposure through working in the wood working industry. This factor tends to increase IPF risk with an odds ratio of 1.56 (95% CI 0.87–2.82, p = 0.14, I^2^ = 38%), which was below statistically significant level (Fig. 3C, Supplementary Table S3).

Four studies (1631 subjects) investigated exposure through working in the textile industry. This included work involving manufacturing or repairing of textiles. This factor did not increase IPF risk significantly with an odds ratio of 1.08 (95% CI 0.64–1.82, p = 0.76, I^2^ = 0%) (Fig. 3D, Supplementary Table S3).

### Smoking and the risk of IPF

**Figure 4:**
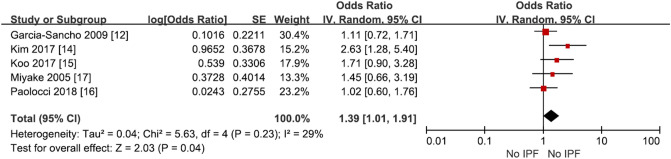
Risk of IPF in ever smoker compared with never smoker.

Among the studies, Awadalla et al. was excluded because it was a smoking-matched case–control study. Meta-analysis showed that smoking increased IPF risk with an odds ratio of 1.38 (95% CI 1.09–1.74, p = 0.008, I^2^ = 10%), which was statistically significant (Fig. 4, Supplementary Table S4).

## Discussion

From previous case-controls studies, we found that metal dust^6,7,9,15–17^ increased the risk of IPF and wood dust^6,7,11,13^ increased IPF risk with statistical significance. Additionally, the exposure to livestock like cattle and birds, livestock feed, pesticides, mold, soil dust, stone dust, stone polishes, and smoke increases IPF prevalence.

On our analysis metal dust, wood dust and pesticide increased the risk of IPF. From the previous case control studies with metal dust and IPF some studies had significant relationship with disease but some other studies did not^11,12,14^. A cohort study of United Kingdom engineering company, increased proportional mortality increased in relation to the duration of metal-working^5^. Metal dust and metal related fumes can deposit in the lung by inhalation or ingestion of the particles and interfere with the pulmonary immune system but specific pathogenesis is not known^20^.

Although a recent, informal meta-analysis, conducted by the international pulmonology conference, meaningful results were found about IPF risk on exposure to the wood dust, metal dust, silica dust, agriculture dust and vapors, gas, dust or fumes^19^. In our study, agricultural dust was not included because only occupational and environmental exposure factors that were included in more than four studies were considered, and silica was excluded because silica was already widely known with silica related lung disease.

Unlike previous studies, our meta-analysis showed statistically significant increase in IPF risk on pesticide exposure (Fig. 2E). Earlier, a case control study about pesticides from Egypt had shown increased risk of IPF^13^. Pesticide exposure can directly and indirectly increase the risk of COPD and asthma^21^. The chemicals can persist in soil for decades. The specific pathogenesis related to ILD is unknown.

A longer occupational exposure period is known to increase IPF risk^16^. Exposure through working in agriculture, livestock, beauty, chemical/petrochemical, woodworking, and steel industries was reported to increase IPF risk previously^9,13,14,16,17^. On meta-analysis, we found statistically significantly increased IPF risk in only agricultural workers. Additionally, the risk of IPF increased through working in building, woodworking, and textile industries, but did not reach statistically significant level.

Only two of the seven included studies showed that individuals’ smoking history statistically significantly increased the risk of developing IPF, but when meta-analysis was conducted, we found that smoking increased IPF risk with an odds ratio of 1.38 (Fig. 4, Supplementary Table S4).

Smoking has been found to increase the risk of IPF in several studies^9,14,16,22^. Studies to date suggest that the increased oxidative stress caused by smoking affects IPF progression in former and current smokers compared to non-smokers^23^. In a study conducted using a population-based registry in Sweden, the risk of IPF increased with an odds ratio of 2.10 (95% CI 1.20–3.68) when subjects smoked for 10–19 pack years and with an odds ratio of 2.25 (95% CI 1.26–4.02) when subjects smoked more for than 20 pack years. Dose-dependent increase was reported for smoking as a risk factor for IPF^23^. Our study also confirmed that the risk of IPF increased in smoker compared to never smoker from meta-analysis on case–control studies.

There were several limitations in this study. First, recall bias may be a limitation of this study. Because the subject’s occupational and environmental risk factors were collected retrospectively, the quality of information may deteriorate because they rely on recall. In four studies, the questionnaire was minimized by direct questionnaire by specialized researchers like occupational environment experts. But remain studies were conducted by the patient himself or herself. Second, a quantitative analysis considering the degree and frequency of exposure would be more informative when investigating risk factors, rather than a simple exposure analysis. However, such an analysis could not be conducted in this study. In terms of occupation type exposure, the actual amount, duration, and frequency of risk factor exposure during the period of exposure in a particular job type was neither conducted nor comparatively analyzed. Third, although studies from various countries are included, they did not have national representation because each study covers a specific region of the country. Also, mainly in the studies in the United States, some European countries, and Asia (where only Japan and Korea are included), racial differences may not be reflected. Fourth, as the diagnostic definition of IPF has been changing for decades. Heterogeneity among the included cases may exist due to the development of imaging technology which may have affected the diagnosis. The first international guidelines^24^, based on expert opinions on the diagnosis and treatment of IPF, were published in 2000 and evidence-based revised new treatment guidelines have been published in 2011, integrating the patient’s clinical symptoms, pathogenesis, and natural course^25^. Later, as new drugs for IPF treatment were developed focusing on early treatment and diagnosis, the new IPF diagnostic criteria, complemented with high-resolution computed tomography imaging and related pathological findings, were presented in 2018^26,27^. This study includes about 30 years of research from 1990 to recent studies. To minimize of misdiagnosis and overdiagnosis of IPF, case control studies that had diagnosed IPF mainly based on chest X-ray were removed^6,7,10^. Finally, in some studies, the control group was not a healthy adult control group. Inclusion of patients with respiratory diseases, such as acute bacterial pneumonia, chronic obstructive pulmonary disease, lung cancer, and pulmonary tuberculosis other than IPF may have affected the interpretation of the results. The effect of smoking as a multiplicative risk for the development of IPF cannot be omitted.

In conclusion, meta-analysis of patient-control studies revealed that exposure to pesticides, metal dust, and wood dust increases the risk of IPF. Additionally, the risk of IPF was more in agricultural workers. Lastly, smoking also increased the risk of IPF.

## Methods

### Searching strategy

The Patient populations, Intervention, Comparison, Outcomes (PICO) of this study are as follows:

P: Adult, IPF cases.
I: Environmental and occupational exposure, occupation.
C: Non-IPF controls.
O: Risk of IPF depending on exposure to each factor.

This study was conducted according to the systematic literature review reporting guidelines of the Preferred Reporting Items for Systematic Reviews and Meta-Analysis (PRISMA)^28^. Medline, Embase, and the Cochrane Library were searched for papers published by March 2020 using the Ovid interface. The search terms were “Idiopathic Pulmonary Fibrosis” [ALL] OR “Idiopathic Pulmonary Fibroses” [ALL] OR “Cryptogenic Fibrosing Alveolitis” [ALL] OR “Cryptogenic Alveolitides” [ALL] OR “Idiopathic Fibrosing Alveolitis” [ALL] OR “Idiopathic Fibrosing Alveolitides” [ALL] OR “Usual Interstitial Pneumonitis” [ALL] OR “Usual Interstitial Pneumonitides” [ALL] OR “Usual Interstitial Pneumonia” [ALL] OR “Usual Interstitial Pneumonias” [ALL]. The studies not recorded in the databases but existing in previous meta-analysis studies were directly searched and added. Additional details are listed in Supplementary Table 1.

### Inclusion and exclusion criteria

Two investigators independently selected the studies after confirming the title and abstract according to the inclusion and exclusion criteria of this study. Duplicate papers were excluded.

The inclusion criteria were: (1) study on adult population over 18 years of age; (2) IPF diagnosis criteria based on the clinician’s judgment of the patient’s symptoms, clinical findings, and imaging findings (histological diagnosis may not have been necessarily included in the diagnosis); (3) categorization of the surveyed jobs or occupational and environmental exposure factors that could lead to risk of IPF. The survey methods for occupational and environmental exposure factors included any method that is systematic and planned, ranging from self-reporting by mail or telephone to face-to-face surveys with experts. Additionally, the effect of cigarettes was analyzed by studying individuals who had smoked in the past or who are currently smoking.

Reviews, letters, editorials, case reports, studies on animals or children, theoretical studies on the medical system itself, revisions after the medical system were introduced, papers or papers not related to the research purpose, papers written in languages other than English were excluded. Additionally, studies that only focused on known risk factors, such as asbestosis, coal worker’s pneumoconiosis, and silicosis, were excluded.

### Evaluation of paper quality

Quality evaluation was conducted for papers that met the inclusion criteria, which was quantitatively evaluated using Risk of Bias Assessment Tool for Nonrandomized Studies (RoBANS)^29^. This tool includes 6 items, including selection of participants, confounding variables, exposure measurement, outcome assessment blinding, incomplete outcome data, and selective reporting. Each item was rated as “high”, “low”, and “uncertain”; 0 for “low”, + 1 for “uncertain”, and + 2 for “high”. On summation, a score of 1 or less meant the paper was of high quality; 2–3, of moderate quality; 4 or more, low in quality.

### Extraction of data

Authors, publication year, location of study, multi-center study, research method, number of experimental and control groups, age, sex, and smoking status were extracted and summarized for the finally included papers.

The number of exposure factors examined in each study was varied. Among them, if more than four studies investigated a common exposure factor, that exposure factor was analyzed. Finally, our analysis was conducted on five exposure factors.

We analyzed four occupations types which were included in four or more studies. Five of the 12 studies included occupational classification in the case–control group^9,13,14,16,17^. Among them, researchers such as Kim and Miyake conducted research according to the Korean Standard Classification of Occupations and Japanese Standard Occupational Classification standards, respectively. The analyzed occupations were classified in each study according to the classification criteria set by the researchers.

The individual's smoking history was classified into two groups, the smoking group including both past and present smoking, and the non-smoking group who had never smoked.

### Statistical analysis

We used Review Manager Version 5.3 (The Cochrane library, Oxford, UK) was used. To minimize the influence of other variables as much as possible, the unadjusted odds ratio value was used. When raw data were presented in the paper, the unadjusted odds ratio was calculated using the presented values. If multiple adjusted odds ratios were presented, the odds ratio values corresponding to the same criteria were used after consultation between authors. Statistical meta-analysis was then performed using the extracted ratio values.

In the main analysis, the occupational and environmental exposure factors included were five types of metal dust, wood dust, stone/sand dust, textile dust, and pesticides. The occupation types included were construction, agriculture, woodworking, and textile. Additionally, the relationship between the individual's smoking history and disease was investigated.

To evaluate statistical heterogeneity in each study, I^2^ test of Higgins was calculated with a 95% confidence interval (CI). Statistical heterogeneity was considered low if I^2^ value was less than 25%, moderate if it was between 25 and 50%, high if it was between 50 and 75%, and very high if it was more than 75%.

After obtaining the odds ratios of each factor, the pooled effect size was estimated using the inverse variance weighted method^30^. The 95% confidence interval and weight are presented as a forest plot.

Sensitivity analysis was conducted to exclude studies of low quality or which included specific conditions or characteristics. If more than 10 studies included in the analysis, a statistical analysis of the asymmetry was performed using Egger’s test to confirm the publication error, and a visual analysis of the asymmetry was performed using a funnel plot.

## Supplementary Information

Supplementary Information.

## Data Availability

The datasets generated during the current study are available from the corresponding author on reasonable request.
