# Supplementary material for: Occupational and environmental risk factors of idiopathic pulmonary fibrosis: a systematic review and meta-analyses
Source: Sci Rep. 2021 Mar 2;11:4318. doi: 10.1038/s41598-021-81591-z (PMC7925580; doi:10.1038/s41598-021-81591-z)
Supplement: Supplementary file 1 — Supplementary Information. [file 41598_2021_81591_MOESM1_ESM.docx]

**Occupational and environmental risk factors of idiopathic pulmonary fibrosis: a systematic review and meta-analyses**

Yeonkyung Park^1,†^, Chiwon Ahn^2,†^, Tae-Hyung Kim^3,*^

^1^Division of Pulmonary and Critical Care Medicine, Department of Internal Medicine, Veterans Health Service Medical Center, Seoul, South Korea.

^2^Department of Emergency Medicine, Chung-Ang University Hospital, Seoul, South Korea

^3^Division of Pulmonary and Critical Care Medicine, Department of Internal Medicine, Hanyang University Guri Hospital, Guri, South Korea

^†^Park and Ahn contributed equally to this work.

^*^**Corresponding author**: Tae-Hyung Kim, M.D., Ph.D.

Division of Pulmonary and Critical Care Medicine, Department of Internal Medicine, College of Medicine, Hanyang University, Seoul, South Korea

Hanyang University Guri Hospital, Guri, South Korea

153, Gyeongchun-ro, Guri-si, Gyeonggi-do, 11923, South Korea

Tel: +82-31-560-2240

Fax: +82-31-553-7369

E-mail: drterry@hanyang.ac.kr


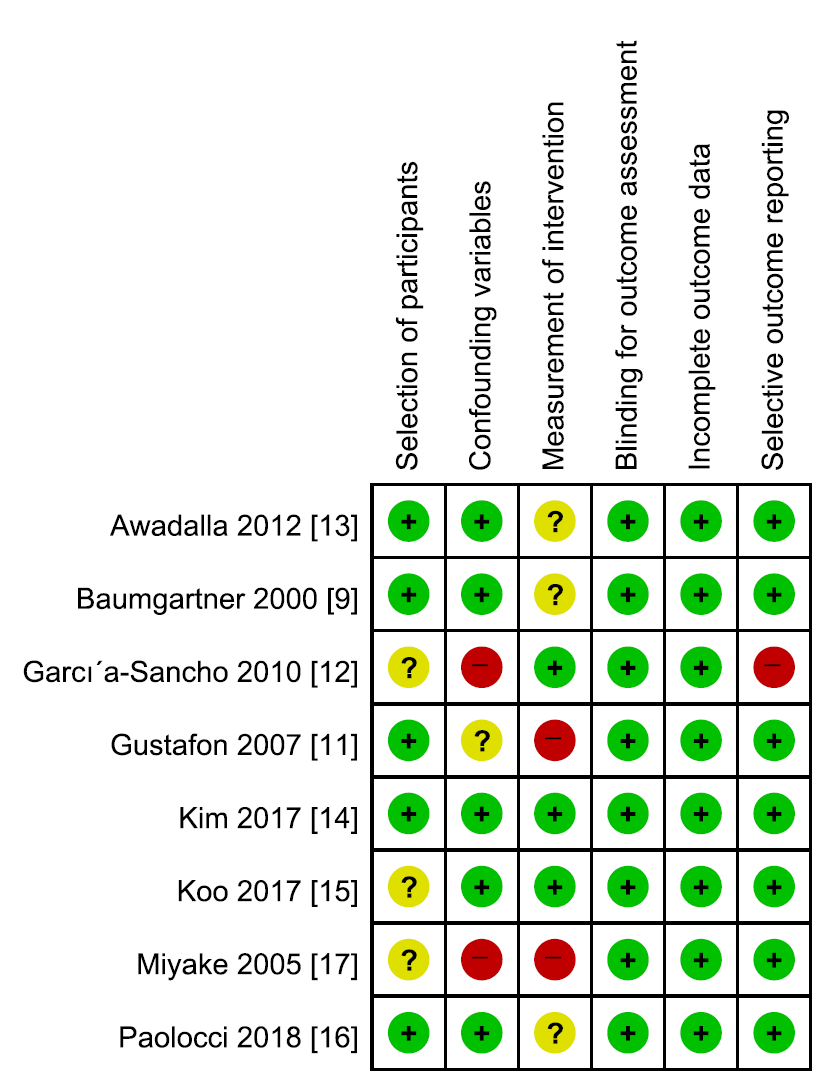


Supplementary Figure 1. The Cochrane Collaboration’s tool for assessing risk of bias

Red (-); high in risk of bias, green (+); low in risk of bias, yellow (?); unclear in risk of bias

Supplementary Table 1. Search Strategy

| Database (n=2,851) | Search term |
| --- | --- |
| Medline | 1) exp lung fibrosis/ (69,063)  2) (idiopath* and pulmonary fibros*).ti,ab,kw. (15,451)  3) exp fibrosing alveolitis/ (23,723)  4) (fibrosing alveoliti* and (cryptogen* or idiopath*)).ti,ab,kw. (506)  5) exp interstitial pneumonia/ (15,330)  6) (interstitial and (pneumonitis* or pneumonia)).ti,ab,kw. (18,929)  7) or/1-6 (88,657)  8) exp risk/ or exp risk factor/ (2,432,167)  9) risk factor*.ti,ab,kw. (867,629)  10) 8 or 9 (2,630,174)  11) exp exposure/ or exp occupational exposure/ or exp environmental exposure/ (607,638)  12) ((occupation* or environment*) and exposure).ti,ab,kw. (185,576)  13) 11 or 12 (696,639)  14) 7 and 10 and 13 (1,614)  15) limit 14 to english language (1,413) |
| Embase | 1) exp Idiopathic Pulmonary Fibrosis/ (4,961)  2) (idiopath* and pulmonary fibros*).ti,ab,kw. (8,364)  3) (fibrosing alveoliti* and (cryptogen* or idiopath*)).ti,ab,kw. (404)  4) exp Lung Diseases, Interstitial/ (55,174)  5) (interstitial and (pneumonitis* or pneumonia)).ti,ab,kw. (11,886)  6) or/1-5 (69,590)  7) exp RISK/ or exp RISK FACTORS/ (1,185,586)  8) (risk or factor*).ti,ab,kw. (4,505,275)  9) 7 or 8 (4,896,161)  10) exp OCCUPATIONAL EXPOSURE/ or exp ENVIRONMENTAL EXPOSURE/ (296,126)  11) ((occupation* or environment*) and exposure).ti,ab,kw. (143,824)  12) 10 or 11 (392,407)  13) 6 and 9 and 12 (1,735)  14) limit 13 to english language (1,423) |
| Cochrane library | 1) Idiopathic Pulmonary Fibrosis [MeSH] OR idiopathic pulmonary fibrosis OR fibrosing alveolitis OR Lung Diseases, Interstitial [MeSH] OR interstitial pneumonitis OR interstitial pneumonia (2,323)  2) Risk [MeSH] OR Risk Factors [MeSH] OR risk factor* (103,223)  3) Occupational Exposure [MeSH] OR Environmental Exposure [MeSH] OR occupational exposure OR environmental exposure (6,131)  4) 1 and 2 and 3 (15) |

Supplementary Table 2. Distribution of subject exposure to occupational and environmental risk factors in included studies between IPF cases and non-IPF controls

| Study | Metal dust | | Wood dust | | Stone/Sand dust | | Textile dust | | Pesticide | |
| --- | --- | --- | --- | --- | --- | --- | --- | --- | --- | --- |
|  | IPF | non-IPF | IPF | non-IPF | IPF | non-IPF | IPF | non-IPF | IPF | non-IPF |
| Awadalla 2012 [13] | 17 (8.5) | 15 (0.7) | 23 (11.0) | 9 (4.4) | 14 (7.0) | 14 (6.8) | 10 (5.0) | 14 (6.8) | 17 (8.5) | 7 (14.0) |
| Baumgartner 2000 [9] | 25 (10.0) | 29 (5.9) |  |  | N/A**^**^** | N/A**^**^** | 20 (8.1) | 25 (5.1) | 8 (3.2) | 11 (2.2) |
| Gustafon 2007 [11] | 25 (18.0) | N/A**^*^** | 22 (16.0) | N/A**^*^** |  |  | 10 (7.1) | 45 (5.9) |  |  |
| Kim 2017 [14] | 9 (13.0) | 6 (8.6) | 7 (10.0) | 6 (8.6) | 10 (14.0) | 1 (1.4) | 4 (5.7) | 2 (2.9) | 15 (21.0) | 7 (10.0) |
| Koo 2017 [15] | 21 (27.0) | 9 (12.0) | 6 (7.7) | 3 (3.9) |  |  |  |  |  |  |
| Miyake 2005 [17] | 12 (12.0) | 1 (1.7) |  |  | 11 (11.0) | 4 (6.8) |  |  | 6 (5.9) | 2 (3.4) |
| Paolocci 2018 [16] | 9 (13.0) | 9 (33.0) |  |  |  |  |  |  |  |  |

N (%), N; numbers of cases and controls exposed to each risk factors, %: proportion of cases and controls exposed to each risk factors; N/A: not available; blank: study not included in analysis of each risk factor; If raw data are provided, calculated unadjusted OR was used for meta-analysis.

**^*^** adjusted OR with sex, year of diagnosis, birth year, smoking was used in meta-analysis

**^**^** adjusted OR with age, smoke was used in meta-analysis.

Supplementary Table 3. Distribution of jobs in included studies between IPF cases and non-IPF controls

| Study | Building construction and demolition workers | | Farming or agriculture workers | | Carpentry and wood workers | | Textile making workers | |
| --- | --- | --- | --- | --- | --- | --- | --- | --- |
|  | IPF | non-IPF | IPF | non-IPF | IPF | non-IPF | IPF | non-IPF |
| Awadalla 2012 [13] | 11 (5.5) | 14 (6.8) | 42 (20.9) | 35 (17.1) | 22 (10.9) | 9 (4.4) | 9 (4.5) | 9 (4.4) |
| Baumgartner 2000 [9] | 10 (4.0) | 18 (3.7) | 62 (25.0) | 95 (19.3) | 27 (10.9) | 44 (9.0) | 4 (1.6) | 5 (1.0) |
| Kim 2017 [14] | 6 (8.6) | 3 (4.3) | 16 (22.9) | 7 (10.0) | 2 (2.9) | 6 (8.6) | 3 (4.3) | 3 (4.3) |
| Miyake 2005 [17] | 11 (10.8) | 5 (8.5) |  |  |  |  |  |  |
| Paolocci 2018 [16] | 14 (20.3) | 26 (9.4) | 30 (43.5) | 51 (18.4) | 6 (8.7) | 15 (5.4) | 9 (13.0) | 36 (13.0) |

N (%), N: numbers of IPF subjects in each job groups; %: Proportion of IPF subjects in each job groups; N/A: not available

Supplementary Table 4. Distribution of ever smoker in included studies between IPF cases and non-IPF controls

| Study | IPF | non-IPF |
| --- | --- | --- |
| Garcia-Sancho 2010[12] | 44(45.4) | 240(42.9) |
| Kim 2017 [14] | 53(75.7) | 38(54.2) |
| Koo 2017 [15] | 52(66.6) | 42(53.8) |
| Miyake 2005 [17] | 84(82.3) | 45(76.3) |
| Paolocci 2018 [16] | 42(60.9) | 167(60.3) |

N (%), N: numbers of ever smokers; %: Proportion of ever smokers in each group; N/A: not available
* Only unadjusted OR was mentioned without numbers of ever smoker in each group
